# Environment‐dependent introgression from *Quercus dentata* to a coastal ecotype of *Quercus mongolica* var. *crispula* in northern Japan

**DOI:** 10.1111/nph.16131

**Published:** 2019-09-23

**Authors:** Teruyoshi Nagamitsu, Kentaro Uchiyama, Ayako Izuno, Hajime Shimizu, Atsushi Nakanishi

**Affiliations:** ^1^ Hokkaido Research Center, Forestry and Forest Products Research Institute Forest Research and Management Organization Sapporo 062‐8516 Japan; ^2^ Department of Forest Molecular Genetics and Biotechnology Forestry and Forest Products Research Institute Forest Research and Management Organization Tsukuba 305‐8687 Japan; ^3^ Greenery Research and Information Center Forestry Research Institute Hokkaido Research Organization Bibai 079‐0198 Japan

**Keywords:** ancestry proportion, coastal stress, double‐digest restriction‐site‐associated DNA (ddRAD) sequencing, heterozygosity, morphological traits, Patterson's *D*, principal component analysis, Weir–Cockerham's *F*_ST_

## Abstract

- Introgression from one species in a specific environment to another may facilitate colonization of the environment by the recipient species. However, such environment‐dependent introgression has been clarified in limited plant taxa.
- In northern Japan, there are two interfertile oak species: *Quercus dentata* (*Qd*) in coastal areas and *Q. mongolica* var. *crispula* (*Qc*) in inland areas. However, at higher latitudes where *Qd* is rare, a coastal *Qc* ecotype with *Qd*‐like traits is distributed in the coastal areas. We distinguished inland *Qc*, coastal *Qc*, and coastal *Qd* populations based on genome‐wide genotypes and multitrait phenotypes and verified introgression from coastal *Qd* to coastal *Qc* using reduced library sequencing.
- Genotypes and phenotypes differed among the populations, and coastal *Qc* was intermediate between inland *Qc* and coastal *Qd*. The ABBA–BABA test showed introgression from coastal *Qd* to coastal *Qc*. In coastal *Qc*, we found various stages of introgression after the first generation of backcross but detected no genomic regions where introgression was enhanced.
- Overall, we show evidence for introgression from a coastal species to an ecotype of an inland species, which has colonized the coastal environment. It remains unclear whether introgressed alleles are selected in the coastal environment.

## Abstract 2

See also the Editorial on this article by https://doi.org/10.1111/nph.16560.

## Introduction

Introgression is the transfer of genes from one taxon to a genomic background of another taxon through hybridization and recurrent backcrossing (Anderson, 1953; Goulet *et al*., 2017). Recombination during backcrossing reorganizes the genome of the recipient taxon with introgressed genes from the donor taxon. The introgressed alleles add new genetic variation to the recipient taxon (Anderson & Stebbins, 1954; Suarez‐Gonzalez *et al*., 2018b). In comparison with other sources of genetic variation, such as standing variation and novel mutation, introgression may be advantageous because the introgressed alleles have been maintained in the environment of the donor taxon (Anderson, 1948). Thus, introgression potentially allows the recipient taxon to rapidly colonize a new habitat that the donor taxon has inhabited (Arnold & Kunte, 2017). Such environment‐dependent introgression has been found in some plants, resulting in the expansion of their habitats and the creation of different ecotypes (Rieseberg *et al*., 2007). Such phenomena have been clarified in limited plant taxa (Whitney *et al*., 2010, 2015; Arnold *et al*., 2016) but have been investigated recently in various plants, including trees (Suarez‐Gonzalez *et al*., 2016, 2018a; Khodwekar & Gailing, 2017).

Oaks are dominant tree species of temperate forests in the northern hemisphere (Denk *et al*., 2017) and are often interfertile among species with ambiguous species boundaries (Petit *et al*., 2004). Introgression between oak taxa, even between deeply divergent lineages (McVay *et al*., 2017), has been found frequently (Guichoux *et al*., 2013; Eaton *et al*., 2015; Sork *et al*., 2016; Kim *et al*., 2018; Ortego *et al*., 2018). Thus, oaks are models for investigating evolutionary consequences of introgression. Oak trees inhabit various types of environment between more stressful habitats, such as coastal, arid, and volcanic areas, and less stressful habitats in mild and productive conditions (Cavender‐Bares, 2019). Therefore, environment‐dependent introgression in oaks has great potential to allow the expansion of their habitats and the creation of different ecotypes.

In northern Japan, there are two species, *Quercus dentata* Thunberg (*Qd*) in coastal areas and *Quercus mongolica* Fischer ex Ledebour var. *crispula* (Blume) H. Ohashi (*Qc*) in inland areas (Matsumoto *et al*., 2009). In northern Hokkaido, the northernmost part of Japan, *Qd* trees are rare because this region is beyond the northern distributional limit of *Qd*. In the region, a coastal *Qc* ecotype with unique traits, which are similar to *Qd* phenotypes and are associated with tolerance to coastal stress, occurs in the coastal area (Aizawa *et al*., 2018). Some taxonomists regard the coastal *Qc* ecotype as a putative hybrid between *Qc* and *Qd* (Ohba, 2006). Nuclear microsatellites demonstrate that an admixture with *Qd* characterizes the genetic background of the coastal *Qc* ecotype (Nagamitsu *et al*., 2019). Thus, environment‐dependent introgression from *Qd* to the coastal *Qc* ecotype is expected but has not yet been confirmed because incomplete lineage sorting of ancestral polymorphism also produces a similar admixture pattern (Muir & Schlötterer, 2004; Lexer *et al*., 2006; Pease & Hahn, 2015).

Genomic studies are effective to distinguish introgression from incomplete lineage sorting of ancestral polymorphism (Goulet *et al*., 2017) and to detect genomic regions where introgression patterns deviate from expectations of neutral genetic processes (Gompert *et al*., 2017; Suarez‐Gonzalez *et al*., 2018b). A whole‐genome sequence of *Quercus robur* Linnaeus is available (Plomion *et al*., 2018), which facilitates genomic studies on oaks. Restriction‐site‐associated DNA (RAD) sequencing is useful for mapping polymorphic sites to the oak reference sequence and determining genotypes at genome‐wide loci (Baird *et al*., 2008). We used double‐digest RAD (ddRAD) sequencing to obtain genome‐wide genotypes (Peterson *et al*., 2012).

Here, we aimed to verify introgression from *Qd* to *Qc* in a coastal environment. Before the verification, we assigned inland *Qc*, coastal *Qc*, and coastal *Qd* populations as nonintrogressed, putatively introgressed, and donor populations, respectively. We used genome‐wide genotypes, multitrait phenotypes, and coastal stress to distinguish these populations. To verify the introgression, we performed the ABBA–BABA test with Patterson's *D* statistic (Durand *et al*., 2011) for the three populations and the outgroup, *Q. robur*, of which the whole‐genome sequence was used as the reference for genotyping. After the verification, we described introgression patterns among individuals and among loci. We expected that nonneutral processes, such as selection in coastal environment, during sufficient backcrossing with *Qc* resulted in genomic heterogeneity in introgression. We examined introgression stages using both ancestry proportion and interancestry heterozygosity (Fitzpatrick, 2013) and genomic heterogeneity using *F*_ST_ outliers (Foll & Gaggiotti, 2008).

## Materials and Methods

### Sampling

**Figure 1:**
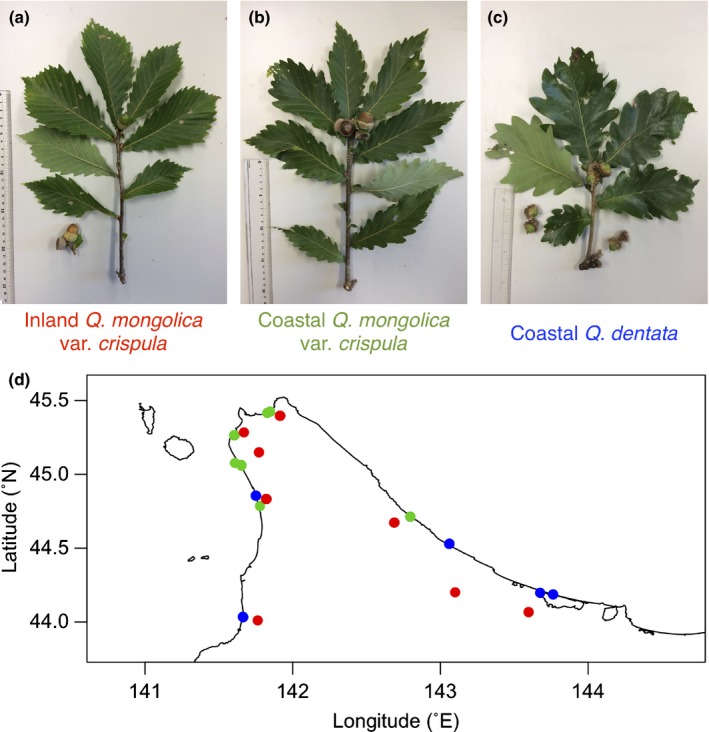
Current‐year shoots and acorns of (a) inland *Quercus mongolica* var. *crispula* (*Qc*), (b) coastal *Qc*, and (c) coastal *Quercus dentata* (*Qd*). (d) Sampling locations (circles) of inland *Qc* (red), coastal *Qc* (green), and coastal *Qd* (blue) in northern Hokkaido.

We collected 16 samples each from the inland *Qc*, coastal *Qc*, and coastal *Qd* trees in northern Hokkaido (Fig. 1). The two species, *Qc* and *Qd*, can hybridize with each other, and their F_1_ hybrids are fertile (Ubukata *et al*., 1999). Though most of the morphological traits of leaves and shoots are continuous between the two species (Ishida *et al*., 2003; Ito, 2009), we identified samples with hairy shoots as *Qd* and those with hairless shoots as *Qc* (Fig. 1a–c). We sampled coastal *Qc* and coastal *Qd* trees from the shore side of oak forests on coastal dunes or cliffs and inland *Qc* trees from broadleaf forests at the foot of mountains (Fig. 1d). The northern limits of the distributional range of *Qd* are located at 44.8°N on the western side and at 44.5°N on the eastern side of Hokkaido (Nagamitsu *et al*., 2019). We sampled coastal *Qd* trees from 44.0°N to the northern limits on both sides of Hokkaido and coastal *Qc* trees from the northern limits to the northernmost part of Hokkaido (Fig. 1d).

In the forests, we sampled trees > 20 m apart from each other to avoid sampling closely related trees. The locations of sampled trees were recorded using GPSMAP 64 (Garmin, Olathe, KS, USA). Two or three branches were collected from sampled trees. Leaves and shoots of collected branches were dried for morphological measurement and preserved in the Hokkaido Research Center, Forestry and Forest Products Research Institute. Fresh leaves were stored at −20°C until DNA extraction.

### Phenotypes and coastal stress

To investigate phenotypes of collected samples, we selected 11 morphological traits of leaves and shoots (Table 1), which are thought to be distinctive between the two species and to be associated with the tolerance to coastal stress (Nagamitsu *et al*., 2019). We measured the morphological traits of sampled trees as described in Supporting Information Notes S1 and Fig. S1.

**Table 1:** Mean values of morphological traits in inland *Quercus mongolica* var. *crispula* (*Qc*), coastal *Qc*, and coastal *Quercus dentata* (*Qd*) samples.

Trait	Mean value in population			Loading
	Inland *Qc*	Coastal *Qc*	Coastal *Qd*	to first PC
Relative leaf width (leaf width/leaf length)	0.519	0.590	0.595	0.271
Relative petiole length (petiole length/leaf length)	0.0253	0.0319	0.0303	0.066
Lateral vain interval (mm) (leaf length/(number of lateral veins + 1))	8.84	11.92	12.98	0.365
Tooth apex angle (°) of serrations	77.7	134.9	156.0	0.393
Density (mm^−2^) of stellate hairs	3.22	7.95	9.06	0.224
Length (mm) of radial filaments of stellate hairs	0.102	0.120	0.286	0.312
Leaf mass per area (mg mm^−2^)	0.757	0.940	0.932	0.292
Shoot diameter (mm)	3.49	5.45	5.57	0.394
Number of axillary buds at leaf scars	7.72	8.98	9.38	0.283
Number of axillary buds at lower stipule scars	3.15	3.60	3.50	0.171
Number of axillary buds at bud scale scars	0.821	2.511	3.271	0.370

Loadings of the traits to the first principal component (PC) of phenotypes are shown.

To describe the leaf shape, four traits were recorded: relative leaf width, relative petiole length, lateral vein interval, and tooth apex angle of serrations. Among the four traits, the two latter traits are important to identify the two species (Ohba, 2006). Stellate hairs on the lower leaf surface vary between the two species, although their functions for the tolerance to coastal stress are unknown (Ishida *et al*., 2003). Both the density and size of stellate hairs were measured. Leaf mass per area and shoot diameter may have physiological functions for adaptation to coastal environment, such as intense light, severe drought, and strong wind (Poorter *et al*., 2009). Salt spray and harsh wind in winter cause the mortality of buds, particularly those in the upper part of shoots (Asai *et al*., 1986). Bud production in the lower part of shoots seems to convey the tolerance to coastal stress because more buds can compensate for the bud mortality. Thus, the number of axillary buds was recorded at leaf scars, at lower stipule scars, and at bud scale scars.

As already mentioned, the bud mortality pattern in shoots reflects coastal stress (Nagamitsu *et al*., 2019). To evaluate coastal stress, we calculated the proportion of flushing buds in the lower part of shoots, *n*/(*m + n*), where *m* and *n* are the numbers of flushing buds in the upper and lower parts of a shoot, respectively (Fig. S1; Notes S1).

### Genotypes

DNA was extracted from stored fresh leaves of each sampled tree using the DNeasy Plant Mini Kit (Qiagen, Hilden, Germany). A ddRAD library was prepared from the 48 samples in the modified methods of Peterson *et al*. (2012). Extracted DNA was digested with *Pst*I and *Sau*3AI restriction enzymes, ligated with Y‐shaped adaptors, and amplified by PCR with the KAPA HiFi polymerase (Kapa Biosystems, Woburn, MA, USA). After PCR amplification with adapter‐specific primer pairs (Access Array Barcode Library for Illumina; Fluidigm, South San Francisco, CA, USA), an equal amount of DNA from each sample was mixed and size‐selected with the BluePippin agarose gel (Sage Science, Beverly, MA, USA). Approx. 450 bp library fragments were retrieved. The library quality was checked using a 2100 Bioanalyzer with a high‐sensitivity DNA chip (Agilent Technologies, Waldbronn, Germany). The detailed methods of library preparation are described in Notes S2. The library was sequenced using an Illumina MiSeq to generate paired‐end reads with a 75 bp length.

The reads obtained were mapped to the reference sequences, 12 pseudomolecules (chromosomes) of oak genome assembly PM1N, of *Q. robur* (Plomion *et al*., 2018). The read mapping and variant calling were conducted using ddocent (Puritz *et al*., 2014). The subsequent filtering procedures are explained in Notes S3. From the variant loci (sites) with various types of polymorphism, we selected sites that were biallelic without indels in both the reference and samples and polymorphic in the samples, with higher sequencing quality and fewer missing genotypes, using VCftools (Danecek *et al*., 2011). We removed sites mapped to the regions of transposable elements determined in *Q. robur* (Plomion *et al*., 2018). In addition, we removed sites that deviated extremely from the Hardy–Weinberg equilibrium within populations using VCftools and sites that potentially had null alleles using GBstools (Cooke *et al*., 2016). Finally, we thinned sites that were tightly linked at < 1 kb intervals.

### Populations

To summarize both phenotypic and genetic variations of the samples obtained, we performed principal component analysis (PCA) using the function *prcomp* of R 3.1.3 (R Core Team, 2016). Some traits, the density and size of stellate hairs and the number of axillary buds at bud scale scars, were skewed. Thus, values of the size, the density + 1, and the number + 1 were log‐transformed. Genotypes at selected ddRAD sites were coded as 0, 1, and 2, which were homozygotes of an allele the same as the reference, heterozygotes of the reference and nonreference (different from the reference) alleles, and homozygotes of the nonreference allele, respectively. The phenotypic values of 11 observed traits and the genotypic values at selected ddRAD sites without missing values were standardized (mean: 0; standard deviation: 1) and applied to the PCA. The contributions of principal components (PCs) to the total phenotypic or genetic variation were obtained. In the PCA of phenotypes, the loadings of the 11 traits to the first PC were calculated.

We distinguished inland *Qc*, coastal *Qc*, and coastal *Qd* populations based on the phenotypic and genetic variations and coastal stress. The samples were plotted on the coordinates of the PCs of genotypes and phenotypes and the proportion of flushing buds in the lower part of shoots. Based on the distribution of the samples, we selected samples representing distinct populations of inland *Qc*, coastal *Qc*, and coastal *Qd*.

### Detecting introgression

The ABBA–BABA test with the Patterson's *D* statistic is useful to detect introgression as distinguishing from incomplete lineage sorting of ancestral polymorphism (Durand *et al*., 2011; Goulet *et al*., 2017). We assumed a phylogenetic relationship (((inland *Qc*, coastal *Qc*), coastal *Qd*), *Q. robur*) according to phylogenetic studies of white oaks (the section *Quercus*) using multiple nuclear loci (Hubert *et al*., 2014; McVay *et al*., 2017; Hipp *et al*., 2018). We calculated the genome‐wide or chromosomal *D* values in this phylogeny as follows:

$$D=\frac{\sum_{i=1}^{n}\left(C_{\mathrm{ABBA}}\left[i\right]-C_{\mathrm{BABA}}\left[i\right]\right)}{\sum_{i=1}^{n}\left(C_{\mathrm{ABBA}}\left[i\right]+C_{\mathrm{BABA}}\left[i\right]\right)}$$

$$C_{\mathrm{ABBA}}\left[i\right]=\left(1-{p_{i}}_{1}\right){p_{i}}_{2}{p_{i}}_{3}\left(1-{p_{i}}_{4}\right),\;C_{\mathrm{BABA}}\left[i\right]={p_{i}}_{1}\left(1-{p_{i}}_{2}\right){p_{i}}_{3}\left(1-{p_{i}}_{4}\right)$$

(*p*_*i*1_, the frequency of a derived allele at site *i* in inland *Qc* (nonintrogressed population); *p*_*i*2_, the frequency of a derived allele at site *i* in coastal *Qc* (putatively introgressed recipient population); *p*_*i*3_, the frequency of a derived allele at site *i* in coastal *Qd* (donor population); *p*_*i*4_, the frequency of a derived allele at site *i* in *Q. robur* (outgroup population); *n*, the number of sites in the whole genome or each chromosome; Eaton *et al*., 2015). A positive *D* value shows that introgression occurred from the donor population to the putatively introgressed recipient population.

The selected samples representing the three populations were used for the ABBA–BABA test. It was ambiguous whether a nonreference allele was a derived one or not because a single reference sequence of *Q. robur* was available. Thus, ambiguous derived alleles were incorporated into the calculation of *D* values using *p*_*i*4_ randomly obtained from a probability distribution (Notes S4).

To obtain genome‐wide and chromosomal *D* estimates, we performed a 1k random sampling of *p*_*i*4_ and then a 1k bootstrap sampling of sites for each sampled *p*_*i*4_ (Notes S4). We obtained the median values and 95% ranges of the 1M bootstrap samples as the estimates and credible intervals, respectively, using the Two functions sample and quantile in R 3.1.3. The probabilities that the genome‐wide *D* values of bootstrap samples were negative and that the chromosomal *D* values of bootstrap samples were less than the genome‐wide estimate were calculated. The probabilities for 12 chromosomes were adjusted to *q* values of the false discovery rate (FDR) in 12 multiple tests using the function *p.adjust* in R 3.1.3. A significantly positive genome‐wide *D* value rejects a null hypothesis that no introgression from coastal *Qd* to coastal *Qc* populations has occurred. A chromosomal *D* value significantly higher than the genome‐wide estimate rejects a null hypothesis that the introgression level of each chromosome is the same as the genome‐wide level.

### Introgression patterns

We described introgression patterns among samples and among sites. We inferred introgression stages using both ancestry proportion *S* (also called hybrid index) and interancestry heterozygosity *H* (Fitzpatrick, 2013) and evaluated genomic heterogeneity in introgression using *F*_ST_ outliers (Foll & Gaggiotti, 2008). The selected samples representing inland *Qc*, coastal *Qc*, and coastal *Qd* populations were used for describing the introgression patterns.

The introgression stages were depicted on the coordinates of *S* and *H*, where *S* is the proportion of alleles descending from the coastal *Qd* population and *H* is the proportion of heterozygous loci with both alleles descending from the inland *Qc* and coastal *Qd* populations (Fitzpatrick, 2013). We estimated *S* and *H* of the samples using the function *HIest* with the options, method = “SANN”, iterations = 10 000, in the package hiest (Fitzpatrick, 2013) in R 3.1.3. The allele frequencies at every site in inland *Qc* and coastal *Qd* populations were obtained from the selected samples representing these populations. To obtain simulated samples in various introgression stages, we created 100 random genotypes at all sites from each of the inland *Qc* and coastal *Qd* populations with the allele frequencies. We simulated random mating between these populations (*Qc* and *Qd*) and obtained 100 simulated samples of their F_1_ hybrids. Similarly, we obtained subsequent generations, F_2_ hybrids and the first generation of backcross with *Qc* (BC_1_ *Qc*) and with *Qd* (BC_1_ *Qd*), and further generations from mating between BC_1_ *Qc* and F_1_, between BC_1_ *Qc* and F_2_, within BC_1_ *Qc*, and between BC_1_ *Qc* and *Qc*. In the simulated samples, *S* and *H* were estimated using HIest in the same method with iterations = 1000. The simulated and observed samples were plotted on the coordinates of *S* and *H* to infer the introgression stages in the coastal *Qc* population.

Wright's *F*_ST_, indicating a difference in allele frequency between populations, represents an indirect measure of gene flow between them. To investigate patterns of introgression across genome‐wide loci, we calculated Weir and Cockerham's estimator of *F*_ST_ at each site in every pair of the three populations using the option ‐weir‐fst‐pop in VCftools 0.1.14 (Danecek *et al*., 2011). To detect *F*_ST_ outlier sites, we surveyed sites with *F*_ST_ values that significantly deviated from an expectation of a neutral genetic process based on the FDR *q* values using bayescan 2.1 (Foll & Gaggiotti, 2008). Outlier sites with high *F*_ST_ values between inland *Qc* and coastal *Qc* populations and low *F*_ST_ values between coastal *Qc* and coastal *Qd* populations indicate enhanced introgression. On the other hand, outlier sites with the opposite combination of *F*_ST_ values indicate restricted introgression. To illustrate patterns of introgression in individual chromosomes, we plotted the three *F*_ST_ values between inland *Qc* and coastal *Qd* populations, between inland *Qc* and coastal *Qc* populations, and between coastal *Qc* and coastal *Qd* populations at individual sites along sequence positions in every chromosome.

## Results

### Genetic and phenotypic variations and coastal stress

We obtained genome‐wide genotypes of 13 inland *Qc*, 15 coastal *Qc*, and 16 coastal *Qd* samples because we omitted three samples of inland *Qc* and one sample of coastal *Qc* with > 30% missing genotypes. Based on ddRAD reads, genotypes at 2772 selected sites in 12 chromosomes were obtained from the 44 samples (Fig. S2a). The intervals of these sites along individual chromosomes varied from 1 kb to 3.2 Mb, and the mean and median intervals were 0.26 Mb and 0.14 Mb, respectively (Fig. S2b). Among the sites, 1347 sites without missing genotypes were used for PCA. The PCA of genotypes resulted in much higher standard deviations of the first PC, which contributed to 12.0% of the total genetic variation, than those of subsequent PCs (< 3.0%; Fig. S3a). The first PC discriminated inland *Qc*, coastal *Qc*, and coastal *Qd* samples, although the subsequent PCs did not (Fig. S3b–f).

In the 44 samples, mean values of 11 measured traits tended to be higher in coastal *Qc* and coastal *Qd* samples than in inland *Qc* samples (Table 1). The PCA of phenotypes of the 11 traits resulted in much higher standard deviations of the first PC (which contributed 40.8% of the total phenotypic variation) than those of subsequent PCs (< 13.6%; Fig. S4a). The first PC separated inland *Qc* samples from coastal *Qc* and coastal *Qd* samples, although the subsequent PCs did not discriminate the three groups of samples (Fig. S4b–f). The lateral vein interval, tooth apex angle, shoot diameter, and number of axillary buds at bud scale scars had high (> 0.35) loadings to the first PC of phenotypes (Table 1).

**Figure 2:**
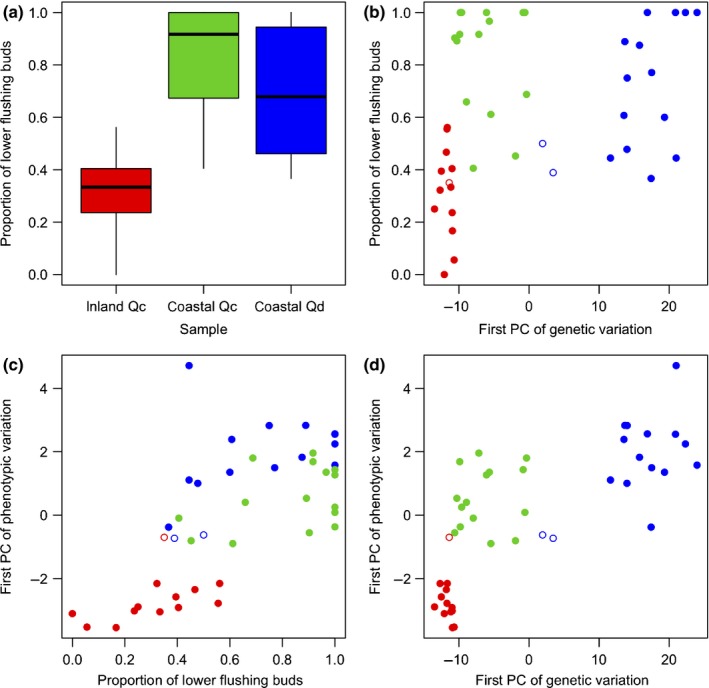
Genetic and phenotypic variations and coastal stress. (a) Box plots of the proportion of lower flushing buds, an index of coastal stress, in inland *Quercus mongolica* var. *crispula* (i*Qc*), coastal *Qc* (c*Qc*), and coastal *Quercus dentata* (c*Qd*) samples. Boxes indicate the first and third quartiles, and whiskers indicate ranges. (b) Relationship between the coastal stress index and the first principal component (PC) values of genotypes. (c) Relationship between the coastal stress index and the first PC values of phenotypes. (d) Relationship between the first PCs of genotypes and phenotypes. Circles indicate i*Qc* (red), c*Qc* (green), and c*Qd* (blue) samples (b–d). Closed circles indicate samples representing i*Qc*, c*Qc* and c*Qd* populations, which are selected for the following analyses, and open circles are removed from the following analyses (b–d).

The proportion of flushing buds in the lower part of shoots was higher in coastal *Qc* and coastal *Qd* samples than in inland *Qc* samples (Kruskal–Wallis test, *P *<* *0.001; Fig. 2a), indicating that stress was higher in the coastal environment. Two coastal *Qd* samples had lower values of the first PC of genotypes than the other coastal *Qd* samples (Fig. 2b,d). One inland *Qc* sample had higher values of the first PC of phenotypes than the other inland *Qc* samples (Fig. 2c,d). These three samples showed intermediate coastal stress between inland and coastal environments and similar phenotypes to coastal *Qc* samples (Fig. 2c). On the coordinates of the first PCs of genotypes and phenotypes, inland *Qc*, coastal *Qc*, and coastal *Qd* samples were clearly separated except for the three samples (Fig. 2d). Thus, we omitted the three samples and selected 12 inland *Qc*, 15 coastal *Qc*, and 14 coastal *Qd* samples representing distinct populations. The first PCs of genotypes and phenotypes differed among these populations (Kruskal–Wallis test, *P *<* *0.001), and the first PCs of coastal *Qc* population were intermediate between those of inland *Qc* and coastal *Qd* populations (Fig. 2d). The coastal *Qc* population was distinct from the coastal *Qd* population in genotypes and distinct from the inland *Qc* population in phenotypes. (Fig. 2d). The coastal *Qc* population differed from the coastal *Qd* population phenotypically and differed from the inland *Qc* population genetically (Wilcoxon test, *P *<* *0.001), but these differences were relatively small (Fig. 2d).

### Introgression

The Patterson's *D* statistic, estimated from allele frequencies at the 2772 ddRAD sites in the 41 selected samples, was significantly positive (*D *=* *0.044, bootstrap *P *<* *0.001), showing introgression from coastal *Qd* to coastal *Qc* populations at genome‐wide loci (Table 2). In the 12 chromosomes, the *D* estimates were positive (≥ 0.007) but were not significantly different from the genome‐wide estimate (FDR *q *>* *0.186; Table 2).

**Table 2:** Genome‐wide and chromosomal estimates of the Patterson's *D* statistic with 95% ranges of bootstrap samples.

	Estimate	Bootstrap sample		*P*‐value
		2.5 percentile	97.5 percentile	
Genome‐wide	0.044	0.026	0.062	< 0.001
Chromosome				
1	0.038	−0.023	0.099	0.568
2	0.092	0.049	0.135	0.015
3	0.052	−0.013	0.115	0.392
4	0.007	−0.068	0.079	0.836
5	0.049	−0.006	0.104	0.452
6	0.048	−0.019	0.115	0.455
7	0.042	−0.013	0.099	0.544
8	0.020	−0.043	0.084	0.783
9	0.014	−0.057	0.083	0.801
10	0.046	−0.017	0.111	0.468
11	0.019	−0.049	0.088	0.774
12	0.039	−0.040	0.117	0.557

*P*‐values of the ABBA–BABA tests that the genome‐wide *D* value is negative and that the chromosomal *D* values are lower than the genome‐wide estimate are also shown.

**Figure 3:**
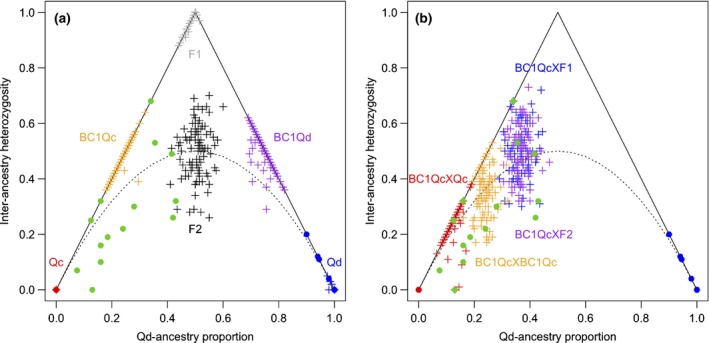
Scatterplots of relationship between the *Quercus dentata* (*Qd*)‐ancestry proportion and the interancestry heterozygosity in observed (circles) and simulated (crosses) samples. Red, green, and blue circles indicate observed samples of inland *Quercus mongolica* var. *crispula* (*Qc*), coastal *Qc*, and coastal *Qd*, respectively. A dotted line indicates an expectation from random mating between inland *Qc* and coastal *Qd* populations. A solid line indicates an expectation from recurrent backcrossing (a) Red, blue, grey, black, orange, and purple crosses indicate simulated samples of inland *Qc* and coastal *Qd* populations, their F_1_ and F_2_ hybrids, and the first generations of backcross with *Qc* (BC_1_ *Qc*) and *Qd* (BC_1_ *Qd*), respectively. (b) Red, orange, blue, and purple crosses indicate simulated samples from mating between BC_1_ *Qc* and *Qc*, within BC_1_ *Qc*, between BC_1_ *Qc* and F_1_, and between BC_1_ *Qc* and F_2_, respectively.

The *Qd*‐ancestry proportion *S* and the interancestry heterozygosity *H* were estimated from genotypes at the 2772 sites in 40 of the 41 selected samples because the estimation failed in one coastal *Qc* sample due to an extremely low maximum log‐likelihood. On the coordinate of *S* and *H*, observed samples of inland *Qc* were located at *S *=* *0 and *H *=* *0, and those of coastal *Qd* were located near *S *=* *1 and *H *=* *0, where simulated samples of their populations (*Qc* and *Qd*) were located (Fig. 3a). Simulated samples were distributed around the expected locations of F_1_ hybrids (*S *=* *0.5, *H *=* *1), F_2_ hybrids (*S *=* *0.5, *H *=* *0.5), and the first generations of backcross with *Qc* (BC_1_ *Qc*:* S *=* *0.25, *H *=* *0.5) and with *Qd* (BC_1_ *Qd*:* S *=* *0.75, *H *=* *0.5; Fig. 3a). Observed samples of coastal *Qc* were located in a wide range on the coordinates (0.06 ≤ *S *≤* *0.44, 0.00 ≤ *H *≤* *0.69; Fig. 3). This range was different from the ranges of simulated samples of F_2_ and BC_1_ *Qc* (Fig. 3a) but was overlapped with those of simulated samples of further generations from mating between BC_1_ *Qc* and F_1_, between BC_1_ *Qc* and F_2_, within BC_1_ *Qc*, and between BC_1_ *Qc* and *Qc* (Fig. 3b). Most of the coastal *Qc* samples were located around the expectation, *H *=* *2*S*(1 − *S*), from random mating between *Qc* and *Qd* populations (Fig. 3).

**Figure 4:**
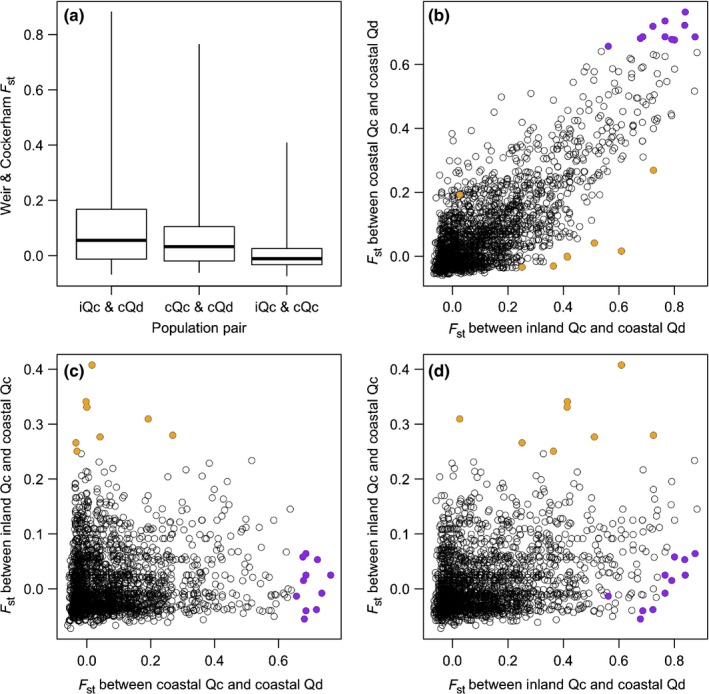
Weir and Cockerham's *F*_ST_ at double‐digest restriction‐site‐associated DNA sites in pairs of three populations, inland *Quercus mongolica* var. *crispula* (*Qc*), coastal *Qc*, and coastal *Quercus dentata* (*Qd*). (a) Box plots of *F*_ST_ values in three pairs of inland *Qc* (i*Qc*), coastal *Qc* (c*Qc*), and coastal *Qd* (c*Qd*) populations. Boxes indicate the first and third quartiles, and whiskers indicate ranges. (b–d) Relationships in *F*_ST_ values between two of the three populations. Orange circles indicate sites with relatively high (> 0.25) *F*_ST_ values between i*Qc* and c*Qc* populations. Purple circles indicate sites with relatively high (> 0.65) *F*_ST_ values between c*Qc* and c*Qd* populations.

The Weir and Cockerham estimator of Wright's *F*_ST_ in every pair of the three populations consisting of the 41 selected samples was obtained from 2633 ddRAD sites, at which *F*_ST_ values were calculated in every pair of the three populations. These *F*_ST_ values were highest between the inland *Qc* and coastal *Qd* populations, intermediate between the coastal *Qc* and coastal *Qd* populations, and lowest between the inland *Qc* and coastal *Qc* populations, in spite of their large variances (Kruskal–Wallis test, *P *<* *0.001; Fig. 4a). No outlier sites were detected in *F*_ST_ between the inland *Qc* and coastal *Qd* populations (FDR *q *≥* *0.074; Fig. S5a), between the inland *Qc* and coastal *Qc* populations (FDR *q *≥* *0.128; Fig. S5b), or between the coastal *Qc* and coastal *Qd* populations (FDR *q *≥* *0.164; Fig. S5c). Nine sites with relatively high (> 0.25) *F*_ST_ values between the inland *Qc* and coastal *Qc* populations (0.251 ≤ *F*_ST_ ≤ 0.408) and 11 sites with relatively high (> 0.65) *F*_ST_ values between the coastal *Qc* and coastal *Qd* populations (0.656 ≤ *F*_ST_ ≤ 0.763) were plotted on the *F*_ST_ distributions (Figs 4b–d, S5). The nine sites showing high genetic differentiation between environments in *Qc* had relatively low *F*_ST_ values (−0.034 ≤ *F*_ST_ ≤ 0.269) between the coastal *Qc* and coastal *Qd* populations (Fig. 4b,c). The 11 sites showing high genetic differentiation between species in the coastal environment had relatively low *F*_ST_ values (−0.055 ≤ *F*_ST_ ≤ 0.064) between the inland *Qc* and coastal *Qc* populations (Fig. 4c,d). Thus, introgression from *Qd* to *Qc* in the coastal environment tended to be enhanced at the nine former sites but restricted at the 11 latter sites, although these sites were not regarded as outliers. The *F*_ST_ values at the 2633 sites in the 12 chromosomes are illustrated in Fig. S6. Three of the nine sites indicating enhanced introgression were aggregated in a genomic region (position 51–53 Mb) of the eighth chromosome (Fig. S6).

## Discussion

The coastal *Qc* ecotype is recognized as a *Qc* population in the coastal environment in northern Hokkaido, which is genetically admixed with *Qd* and is phenotypically similar to *Qd* (Ohba, 2006; Aizawa *et al*., 2018; Nagamitsu *et al*., 2019). This study confirms these properties quantitatively based on genome‐wide genotypes, multitrait phenotypes, and coastal stress. Oaks in the coastal environment are under stress from various factors, such as wind, salinity, drought, and heat (Ciccarelli, 2015). Among these factors, strong wind and salt spray in winter are critical factors that damage terminal buds and inhibit shoot elongation, affecting tree shape and canopy height (Asai *et al*., 1986). Thus, we measured the flushing pattern of buds in the upper and lower parts of shoots as an index of coastal stress. This study demonstrates that the coastal *Qc* ecotype is under coastal stress similar to that of *Qd* in the coastal environment and different from that of *Qc* in the inland environment. Because the flushing pattern of buds is a response of oak traits to environment, not only environmental conditions but also genotypes and phenotypes are involved in the difference in the coastal stress index. As expected from the properties of the coastal *Qc* ecotype, its genotypes and phenotypes were intermediate between those of inland *Qc* and coastal *Qd*. This study reveals that the coastal *Qc* ecotype is distinct from the coastal *Qd* in genotypes and distinct from the inland *Qc* in phenotypes. On the other hand, the coastal *Qc* ecotype shows relatively small differences from the coastal *Qd* in phenotypes and from the inland *Qc* in genotypes.

The relatively large phenotypic variation between inland *Qc* and coastal *Qc* in spite of the relatively small genetic variation between them may be partly due to phenotypic plasticity responding to their different environments. Oaks are known to have phenotypic plasticity in response to various environmental conditions related to coastal stress, such as temperature, drought, and light (Ramirez‐Valiente *et al*., 2010; Cavender‐Bares, 2019). The observed phenotypic variation was mainly associated with leaf shape, shoot diameter, and bud production. Our previous study shows that these traits depend not only on the proportion of admixture with *Qd* estimated from nuclear microsatellites but also on the proportion of lower flushing buds, which is the same as measured in this study (Nagamitsu *et al*., 2019). Thus, the findings suggest both genetic (admixture with *Qd*) and environmental (coastal stress) effects on the phenotypes of the coastal *Qc* ecotype. The previous study also shows interactions between the genetic and environmental effects, suggesting different reaction norms to a coastal environment among trees with different admixture proportions (Nagamitsu *et al*., 2019). To discriminate the genetic and environmental effects and their interactions, comparison of traits of trees planted in common gardens is necessary (Cavender‐Bares & Ramírez‐Valiente, 2017). In such an experiment, it is required that trees are sampled from inland *Qc*, coastal *Qc*, and coastal *Qc* populations, and that common gardens are located in inland and coastal environments. In the experiment, survival, growth, and reproduction of the trees can be evaluated. These approaches are essential to elucidate the adaptation of the coastal *Qc* ecotype to the coastal environment (Hedrick, 2013; Suarez‐Gonzalez *et al*., 2018b), however, which is beyond the scope of this study.

Although we distinguished the three populations, we found some samples with intermediate genotypes and phenotypes among the populations. The findings suggest that genetic and phenotypic variations in the fields are more continuous than those in the samples of this study, forming clines among the populations. A cline from one taxon to another through their hybrids is called a hybrid zone (Harrison, 1990). Hybrid zones are often accompanied by environmental gradients, and the parental taxa of the hybrids inhabit different environments (Barton & Hewitt, 1985). Fundamental theories and useful tools have been developed to investigate hybrid zones (Gompert *et al*., 2017). Coastal oak forests that include both *Qc* and *Qd* trees in northern Japan are known to form hybrid zones between *Qd* on the coastal side and *Qc* on the inland side (Matsumoto *et al*., 2009). However, the coastal *Qc* ecotype in northern Hokkaido is in a system different from a typical hybrid zone because *Qd* is rare on the coastal side at higher latitudes in this region. The northern distributional limit of *Qd* spatially separates the coastal *Qc* ecotype from *Qd*, which may result in the observed genetic gap between them. Because of these features, population‐based analyses are more suitable for the samples of this study than common tools for hybrid zones, such as genomic cline analyses (Gompert & Buerkle, 2009, 2011). Thus, we defined inland *Qc*, coastal *Qc*, and coastal *Qd* populations as nonintrogressed, putatively introgressed, and donor populations, respectively, to investigate introgression.

Allele sharing between taxa results from both introgression between them and incomplete lineage sorting of polymorphism in their ancestral lineage (Muir & Schlötterer, 2004; Lexer *et al*., 2006; Goulet *et al*., 2017). In the former, alleles that emerged in either taxon transfer between the taxa after their divergence. In the latter, both taxa inherit alleles that emerged in the ancestral lineage before the divergence. We tried to detect introgression as discriminating from incomplete lineage sorting using the ABBA–BABA test with a European white oak species, *Q. robur*, as an outgroup (Durand *et al*., 2011; Pease & Hahn, 2015). Both *Qc* and *Qd* belong to white oaks (Denk *et al*., 2017), and their comprehensive phylogeny has been reconstructed (Hubert *et al*., 2014; McVay *et al*., 2017; Hipp *et al*., 2018). Hipp *et al*. (2018) estimated that East Asian white oaks (the ancestral lineage of *Qc* and *Qd*) and European white oaks (the sister group) diverged 15–20 Ma and that *Q. mongolica* (the species including *Qc*) and the clade including *Qd* diverged *c*. 10 Ma. These estimates indicate similar durations when derived alleles accumulated in *Qd* and the ancestral lineage. Thus, it is feasible that *Qc* and *Qd* share derived alleles by means of introgression as well as incomplete lineage sorting of ancestral polymorphism. The ABBA–BABA test of this study shows introgression from coastal *Qd* to the coastal *Qc* ecotype at genome‐wide loci. Because the ABBA–BABA test assumes that alleles derived from an ancestral lineage are randomly sorted to descendant populations, nonneutral processes that bias this sorting can lead to false‐positive introgression (Martin *et al*., 2015). However, it is unlikely that nonneutral processes, such as selection in a coastal environment, affect genome‐wide loci of the coastal *Qc* ecotype.

Introgression begins with hybridization and proceeds during recurrent backcrossing (Anderson, 1953; Goulet *et al*., 2017). We estimated stages of this process using two parameters: the ancestry proportion and interancestry heterozygosity (Fitzpatrick, 2013). We found various introgression stages after the first generation of backcross (BC_1_) with *Qc* in the coastal *Qc* ecotype. Because subsequent hybrid generations created from random mating are expected to have similar parameters, it is difficult to discriminate different generations after F_2_ hybrids and BC_1_ (Gompert & Buerkle, 2016). Thus, it is unclear how many generations after BC_1_ have passed in the coastal *Qc* ecotype. Not only current hybridization but also ancient introgression is known in white oaks (Sork *et al*., 2016; Kim *et al*., 2018). These examples suggest a possibility that the coastal *Qc* ecotype originated from ancient introgression. Drift or selection decreases the interancestry heterozygosity, leading to fixation of introgressed alleles in the genomic background of either ancestry (Gompert *et al*., 2014). Such fixation was rare in the coastal *Qc* ecotype, and most of the samples were expected to derive from random mating between *Qc* and *Qd* ancestry. These findings do not support that selection of introgressed alleles in a coastal environment and/or drift due to demographic bottlenecks alter the genomic composition of the coastal *Qc* ecotype.

Genomic heterogeneity in introgression was found in European white oaks that experienced secondary contacts (Guichoux *et al*., 2013; Leroy *et al*., 2017). A locus showing enhanced introgression was identified in North American red oaks (the section *Lobatae*) along a soil moisture gradient, suggesting that dry environment selected introgressed alleles at the locus (Lind‐Riehl *et al*., 2014; Khodwekar & Gailing, 2017). In this study, we detected neither chromosomes nor loci deviated from a neutral expectation of introgression. However, we found some loci with a combination of high genetic differentiation within *Qc* between inland and coastal environments and low genetic differentiation within the coastal environment between *Qc* and *Qd*, suggesting enhanced introgression. We also found some loci with the opposite combination, suggesting restricted introgression. Some of the loci showing enhanced introgression were aggregated in a genomic region. These findings imply genomic heterogeneity in introgression, although this study failed to detect outliers. Insufficient numbers of loci and samples in this study may be responsible for the failure. First, it is difficult to detect loci at which introgressed alleles have been sufficiently selected due to their narrow blocks of linkage disequilibrium using a small number of loci; this is because sufficient recombination during the selection reduces the length of the blocks (Gompert *et al*., 2017). Second, it is difficult to detect loci at which introgressed alleles have been insufficiently selected due to their weak signature of selection using a small number of samples.

In conclusion, this study shows introgression from the coastal oak species *Qd* to an ecotype of the inland oak species *Qc*, which colonized the coastal environment outside the distributional range of *Qd*. This system is a typical example of environment‐dependent introgression resulting in the expansion of habitats and the creation of ecotypes. The coastal *Qc* ecotype is a mixture of individuals in various introgression stages after the first generation of backcross. Although genomic heterogeneity in introgression is expected, this study detects no genomic regions deviated from a neutral expectation of introgression. Thus, it remains unclear that introgressed *Qd* alleles are selected in the coastal environment. Further studies are necessary to show whether the introgression in the coastal *Qc* ecotype leads to the adaptation to the colonizing environment.

## Author contributions

TN and HS designed the study. TN and AN collected the samples. KU, AI and TN analyzed the data. TN wrote the manuscript.

## Supporting information

Please note: Wiley Blackwell are not responsible for the content or functionality of any Supporting Information supplied by the authors. Any queries (other than missing material) should be directed to the *New Phytologist* Central Office.

**Fig. S1** Morphological measurements of leaf and shoot traits and coastal stress.
**Fig. S2** Distributions of selected ddRAD sites.
**Fig. S3** Principal component analysis of genotypes.
**Fig. S4** Principal component analysis of phenotypes.
**Fig. S5** Relationships between *q* values of the false discovery rate (FDR) and the Weir and Cockerham's *F*_ST_ between populations.
**Fig. S6** Weir and Cockerham's *F*_ST_ among populations at ddRAD sites in individual chromosomes.

**Notes S1** Morphological measurements.
**Notes S2** Preparation of ddRAD library.
**Notes S3** Variant calling and site filtering.
**Notes S4** Patterson's *D* with ambiguous derived alleles.
