# Supplementary material for: Environment‐dependent introgression from Quercus dentata to a coastal ecotype of Quercus mongolica var. crispula in northern Japan
Source: New Phytol. 2019 Sep 23;226(4):1018–28. doi: 10.1111/nph.16131 (PMC7216917; doi:10.1111/nph.16131)
Supplement: Supplementary file 1 — Fig. S1 Morphological measurements of leaf and shoot traits and coastal stress. Fig. S2 Distributions of selected ddRAD sites. Fig. S3 Principal component analysis of genotypes. Fig. S4 Principal component analysis of phenotypes. Fig. S5 Relationships between q values of the false discovery rate (FDR) and the Weir and Cockerham's F ST between populations. Fig. S6 Weir and Cockerham's F ST among populations at ddRAD sites in individual chromosomes. [file NPH-226-1018-s001.pdf]

Fig. S1; Article title: Environment-dependent introgression from *Quercus dentata* to a coastal ecotype of *Q. mongolica* var. *crispula* in northern Japan
Authors: Teruyoshi Nagamitsu, Kentaro Uchiyama, Ayako Izuno, Hajime Shimizu, and Atsushi Nakanishi; Article acceptance date: 9 August 2019

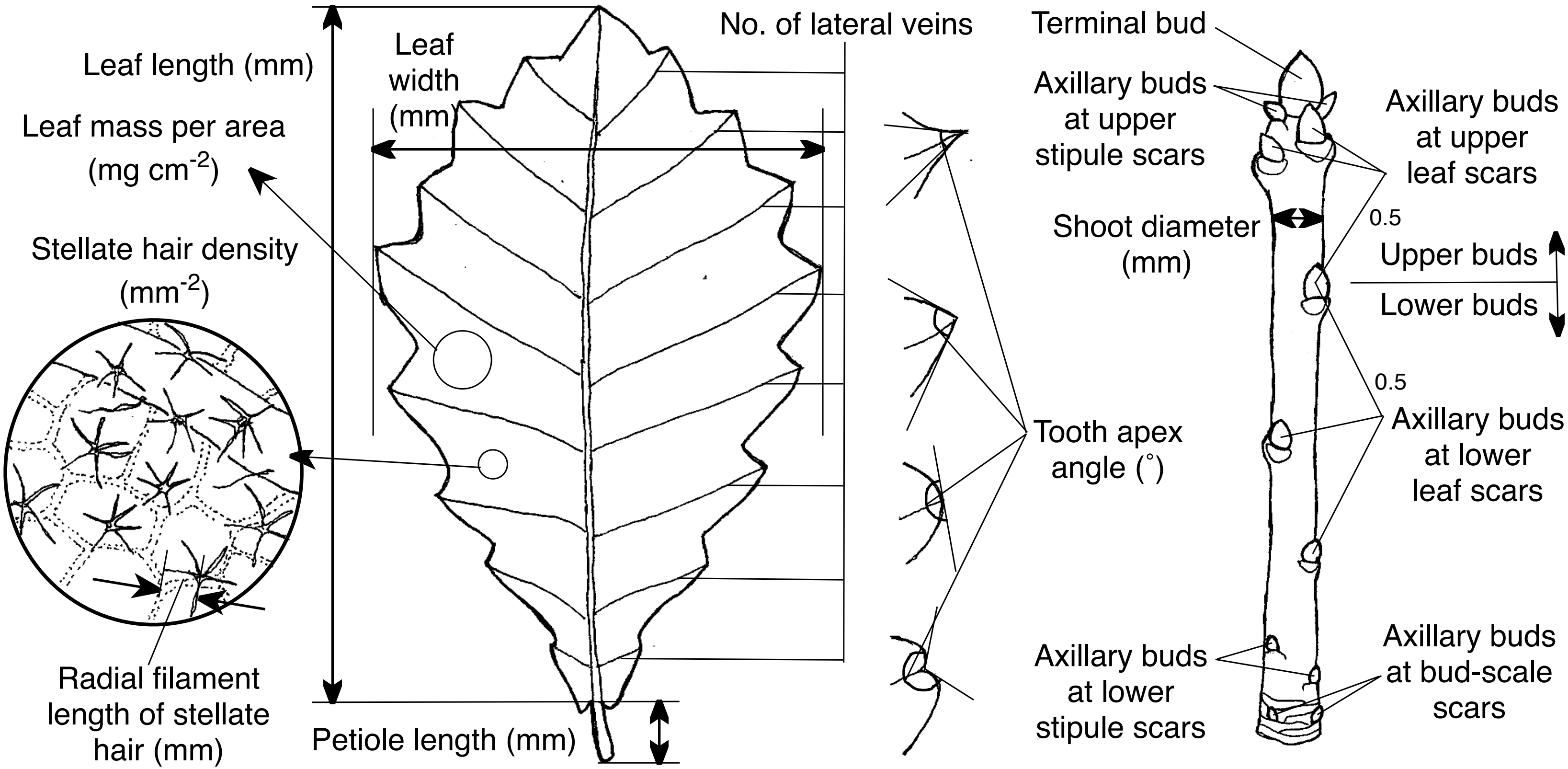

Fig. S1. Morphological measurements of leaf and shoot traits and coastal stress.

Fig. S2; Article title: Environment-dependent introgression from *Quercus dentata* to a coastal ecotype of *Q. mongolica* var. *crispula* in northern Japan
Authors: Teruyoshi Nagamitsu, Kentaro Uchiyama, Ayako Izuno, Hajime Shimizu, and Atsushi Nakanishi; Article acceptance date: 9 August 2019

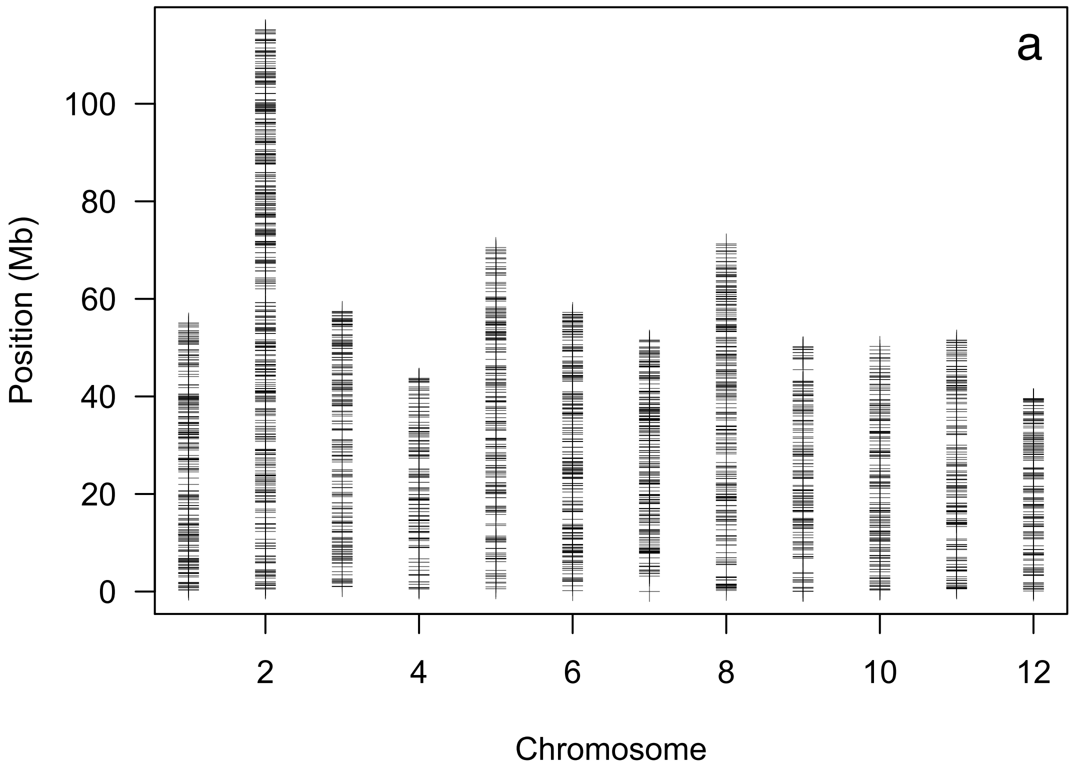

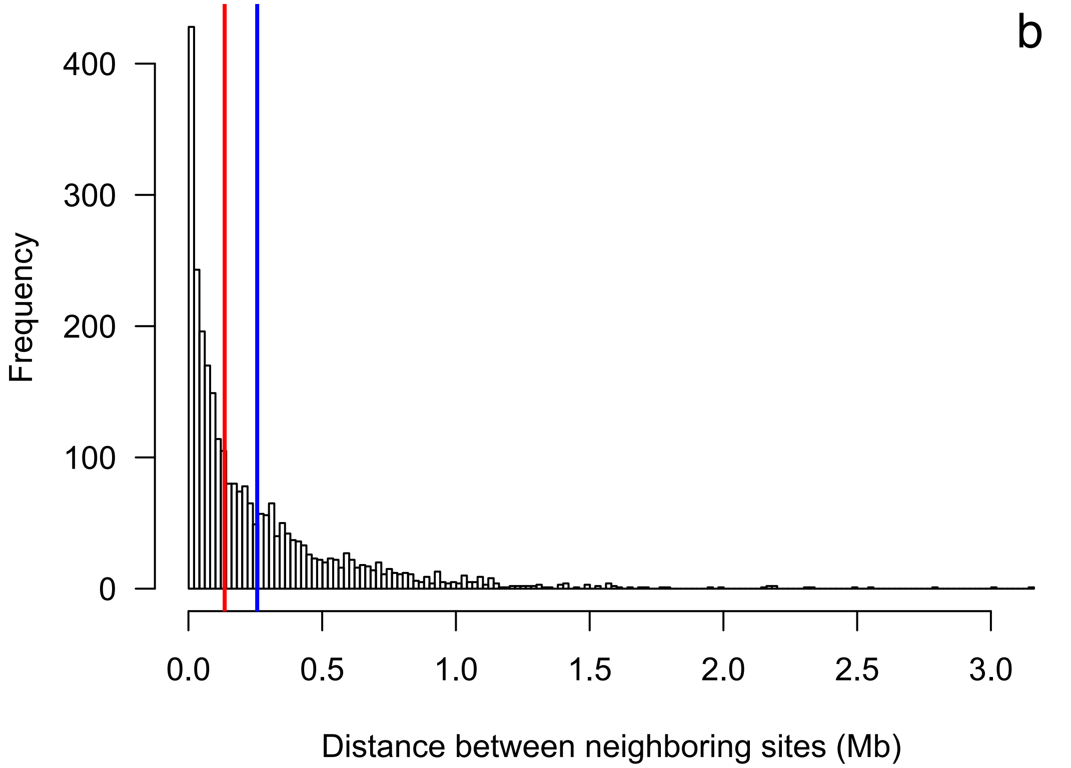

Fig. S2. Distributions of selected ddRAD sites. (**a**) Positions of 2772 ddRAD sites in 12 pseudomolecules (chromosomes). (**b**) Histograms of distances (Mb) between neighboring ddRAD sites in individual chromosomes. Red and blue vertical lines indicate median and mean values, respectively.

Fig. S3; Article title: Environment-dependent introgression from *Quercus dentata* to a coastal ecotype of *Q. mongolica* var. *crispula* in northern Japan
Authors: Teruyoshi Nagamitsu, Kentaro Uchiyama, Ayako Izuno, Hajime Shimizu, and Atsushi Nakanishi; Article acceptance date: 9 August 2019

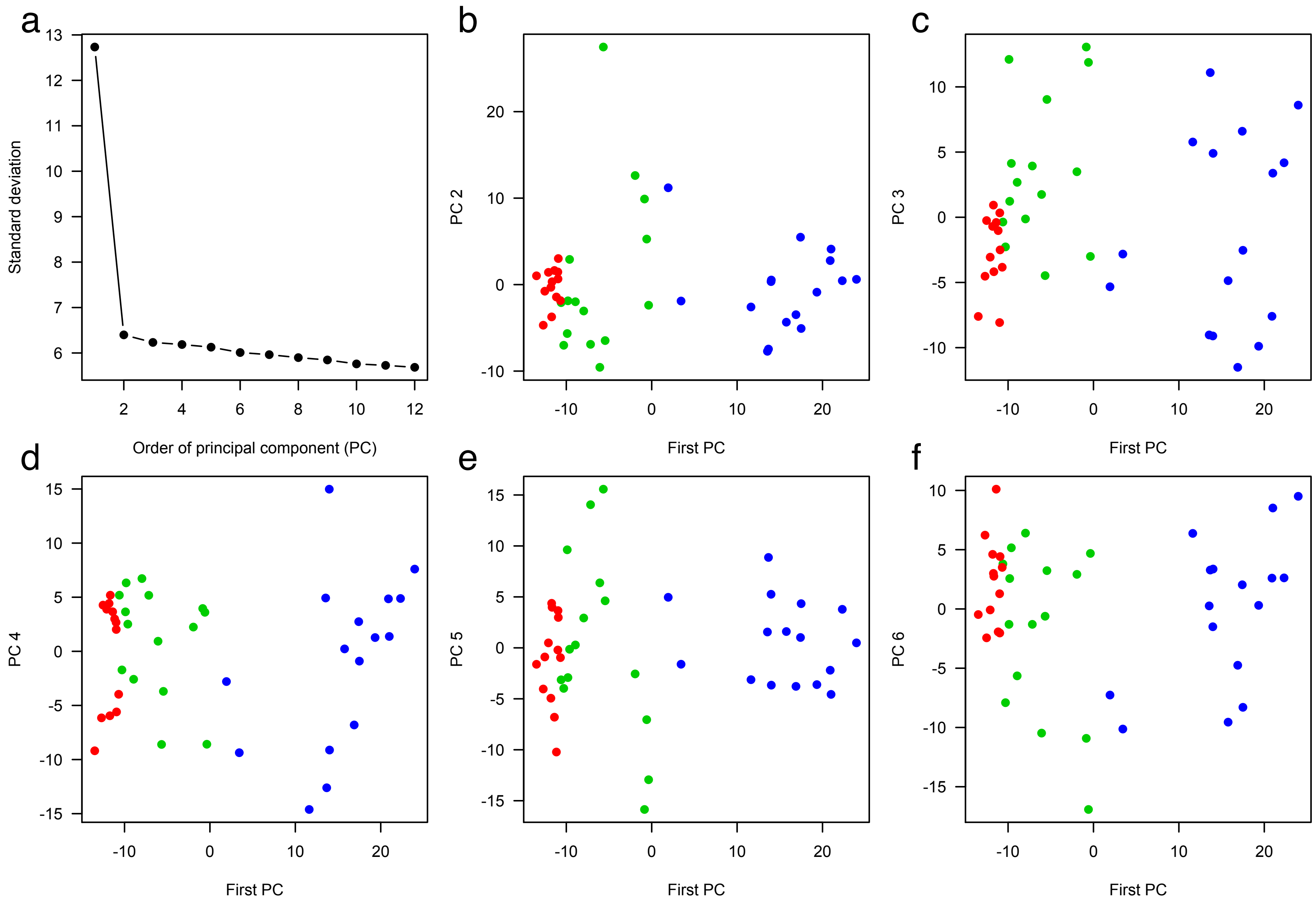

Fig. S3. Principal component analysis of genotypes. (**a**) Standard deviation of principal components (PCs) for 1347 selected ddRAD sites without missing genotypes. (**b–f**) Scatter plots between the first PC and subsequent PCs from the second to sixth orders. Red, green, and blue circles indicate inland *Qc*, coastal *Qc*, and coastal *Qd* samples, respectively.

Fig. S4; Article title: Environment-dependent introgression from *Quercus dentata* to a coastal ecotype of *Q. mongolica* var. *crispula* in northern Japan
Authors: Teruyoshi Nagamitsu, Kentaro Uchiyama, Ayako Izuno, Hajime Shimizu, and Atsushi Nakanishi; Article acceptance date: 9 August 2019

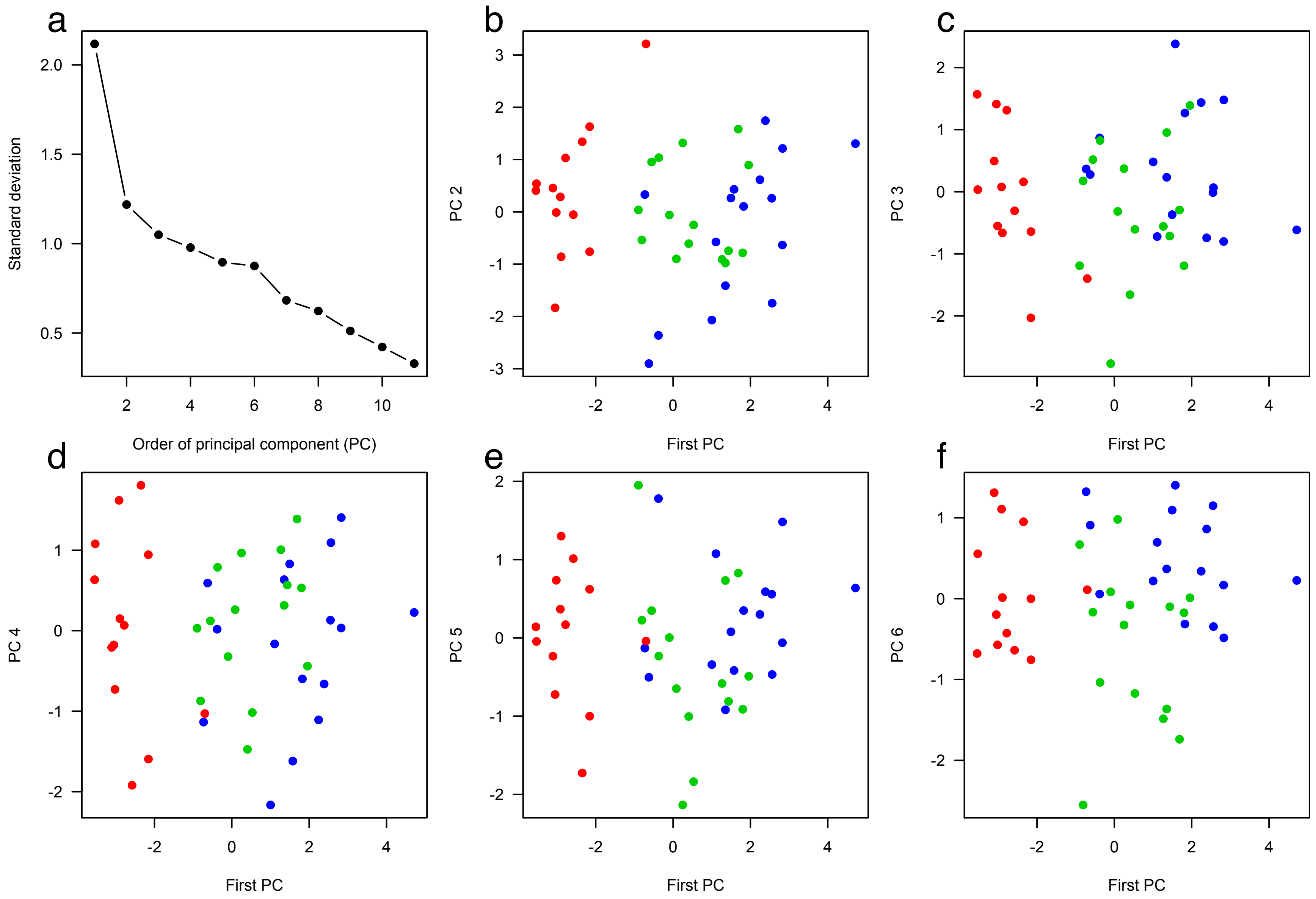

Fig. S4. Principal component analysis of phenotypes. (**a**) Standard deviation of principal components (PCs) for 11 traits. (**b–f**) Scatter plots between the first PC and subsequent PCs from the second to sixth orders. Red, green, and blue circles indicate inland *Qc*, coastal *Qc*, and coastal *Qd* samples, respectively.

Fig. S5; Article title: Environment-dependent introgression from *Quercus dentata* to a coastal ecotype of *Q. mongolica* var. *crispula* in northern Japan
Authors: Teruyoshi Nagamitsu, Kentaro Uchiyama, Ayako Izuno, Hajime Shimizu, and Atsushi Nakanishi; Article acceptance date: 9 August 2019

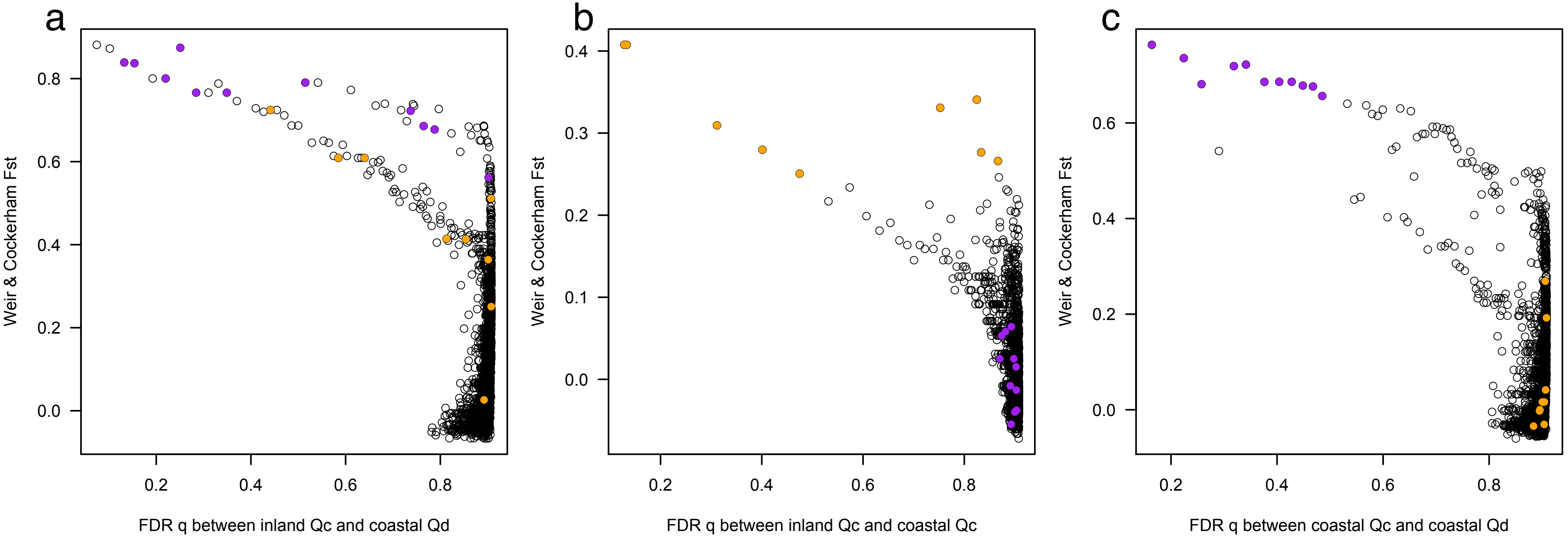

Fig. S5. Relationships between *q* values of the false discovery rate (FDR) and the Weir and Cockerham's F_{ST} between two of three populations, (**a**) inland *Qc* and coastal *Qd*, (**b**) inland *Qc* and coastal *Qc*, and (**c**) coastal *Qc* and coastal *Qd*. Orange circles indicate marginal sites with high (> 0.25) F_{ST} values between inland *Qc* and coastal *Qc* populations. Purple circles indicate marginal sites with high (> 0.65) F_{ST} values between coastal *Qc* and coastal *Qd* populations.

Fig. S6; Article title: Environment-dependent introgression from *Quercus dentata* to a coastal ecotype of *Q. mongolica* var. *crispula* in northern Japan
Authors: Teruyoshi Nagamitsu, Kentaro Uchiyama, Ayako Izuno, Hajime Shimizu, and Atsushi Nakanishi; Article acceptance date: 9 August 2019

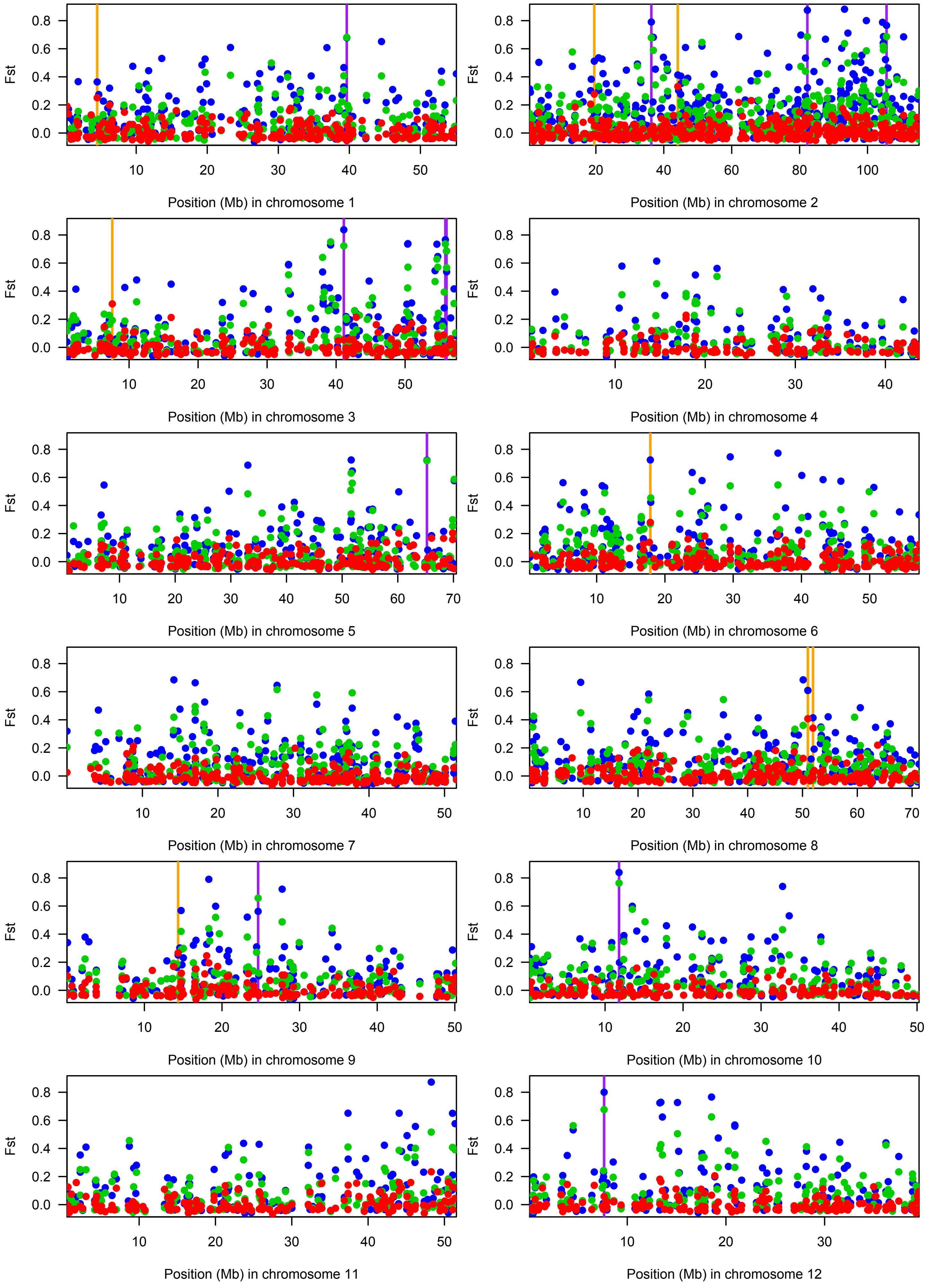

Fig. S6. Weir and Cockerham's F_{ST} among inland *Quercus mongolica* var. *crispula* (*Qc*), coastal *Qc*, and coastal *Q. dentata* (*Qd*) populations at ddRAD sites in individual chromosomes. Blue circles indicate F_{ST} values between inland *Qc* and coastal *Qd* populations, green circles indicate those between coastal *Qc* and coastal *Qd* populations, and red circles indicate those between inland *Qc* and coastal *Qc* populations. Orange vertical lines indicate positions of sites with relatively high (> 0.25) F_{ST} values between inland *Qc* and coastal *Qc* populations. Purple vertical lines indicate positions of sites with relatively high (> 0.65) F_{ST} values between coastal *Qc* and coastal *Qd* populations.
