# Supplementary material for: Environment‐dependent introgression from Quercus dentata to a coastal ecotype of Quercus mongolica var. crispula in northern Japan
Source: New Phytol. 2019 Sep 23;226(4):1018–28. doi: 10.1111/nph.16131 (PMC7216917; doi:10.1111/nph.16131)
Supplement: Supplementary file 2 — Notes S1 Morphological measurements. Notes S2 Preparation of ddRAD library. Notes S3 Variant calling and site filtering. Notes S4 Patterson's D with ambiguous derived alleles. [file NPH-226-1018-s002.pdf]

Notes S1–4

Article title: Environment-dependent introgression from *Quercus dentata* to a coastal ecotype of *Q. mongolica* var. *crispula* in northern Japan

Authors: Teruyoshi Nagamitsu, Kentaro Uchiyama, Ayako Izuno, Hajime Shimizu, and Atsushi Nakanishi

Article acceptance date: 9 August 2019

Note S1. Morphological measurements

Three undamaged leaves were selected from each sampled tree. The length between the apex and base of each leaf, width at the widest part of the leaf, and length of its petiole (mm) were measured, and the lateral veins were counted (Fig. S1). Relative leaf width was calculated from (leaf width)/(leaf length); relative petiole length was calculated from (petiole length)/(leaf length); and the lateral vein interval (mm) was calculated from (leaf length)/(number of lateral veins + 1). The angle of a tooth apex (˚) was measured on a serration at the central part of each leaf (Fig. S1). A leaf disc with a 9 mm diameter was collected from the central part of each leaf (Fig. S1), and its dry weight (mg) was measured after dehydration at 60˚C for 48 h. Stellate hairs on the lower surface of each leaf were observed in a leaf area with a 3.1 mm diameter between lateral veins using a stereo microscope (Fig. S1). The stellate hairs in the area were counted, and the density (mm^{-2}) was obtained (Fig. S1). As the size of stallate hairs, the length of a radial filament (mm) of a typical stellate hair was measured (Fig. S1).

Three undamaged one-year-old shoots were selected from each sampled tree. The diameter of the upper part of each shoot (mm) was measured (Fig. S1). Buds of the shoot were classified as a terminal bud, axillary buds at upper stipule scars, those at leaf scars, those at lower stipule scars, and those at bud scale scars (Fig. S1). Buds in the upper part include the terminal bud, the axillary buds at upper stipule scars, and the upper half of those at leaf scars (Fig. S1). Buds in the lower part include the axillary buds at bud scale scars, those at lower stipule scars, and the lower half of those at leaf scars (Fig. S1). The flushing buds in the upper (m) and lower (n) parts of a shoot were counted. When there was an odd number of axillary buds at leaf scars, a living bud at the central position was divided into the upper and lower parts, and 0.5 was added to both m and n (Fig. S1).

To obtain a trait value of each sample, we averaged the measurements of three leaves or three shoots from each sampled tree.

Note S2. Preparation of ddRAD library

From each sample, 250 ng of the DNA template was double-digested with *Pst*I-HF (10 units per reaction, New England Biolabs Inc., Beverly, MA, USA) and *Sau*3AI (10 units per reaction, New England Biolabs Inc.) at 37°C for 16 h. Each fragmented DNA was purified using the homemade AMPure beads (using Sera-Mag SpeedBeads, Thermo Fisher Scientific, Waltham, MA, USA) and eluted with 25 µL of low TE buffer (pH 8.0).

Purified DNA was then ligated to CS1-tagged and CS2-tagged adapters, consisting of the common sequence tag 1 (CS1) and the common sequence tag 2 (CS2) of Access Array, respectively. The CS1-tagged adapter binds to overhangs generated by *Pst*I, and the CS2-tagged adapter contains overhangs compatible with an *Sau*3AI site. Adapter ligation was performed in a 40µL reaction volume containing 25µL of DNA, 2 µL of the CS1-tagged adapter (100 nM), 4 µL of the CS2-tagged adapter (100 nM), 1 µL of a T4 ligase (1 U/µL, Invitrogen, Carlsbad, CA, USA) and 8 µL of a 5 × T4 ligation buffer (Invitrogen). The reaction was incubated at 25°C for 1 h, and the ligase was then heat-inactivated at 65°C for 30 min.

After the adapter ligation, each DNA sample was purified using AMPure beads and amplified using KAPA HiFi polymerase (Kapa Biosystems, Woburn, MA, USA) with 400 nM primers of the Access Array Barcode Library for Illumina Sequencer 384 (Fluidigm, South San Francisco, CA, USA). The following PCR protocol was used: initial denaturation at 95°C for 5 min; 12 cycles of 95°C for 15 s, 60°C for 30 s and 72°C for 1 min; followed by a final extension period at 72°C for 3 min. The amplified libraries were purified using the AMPure beads and quantified using the Qubit fluorometer with a Quant-iT dsDNA HS Assay Kit (Invitrogen).

An equal amount of DNA from each sample was mixed and size-selected using BluePippin agarose gel (2% agarose cartridge, Sage Science, Beverly, MA, USA) under a “narrow” setting with a mean of 450 bp. After the size selection, each sample was purified using the AMPure beads. The quality, size, and concentration of the pooled libraries were finally determined using the 2100 Bioanalyzer with a high-sensitivity

DNA chip (Agilent Technologies, Waldbronn, Germany), and the library was diluted before template preparation.

Note S3. Variant calling and site filtering

Compressed fastq files of paired-end reads with a 75 bp length of 48 barcoded samples named as X,

```
X.F.fq.gz, X.R.fq.gz
```

were obtained from the Illumina MiSeq. These reads were mapped to sequences of the oak genome assembly PM1N of *Q. robur* in a fasta file (https://urgi.versailles.inra.fr/download/oak/Qrob_PM1N.fa.gz). Mapping and variant calling were conducted using dDocent. Sites in an input vcf file from dDocent were selected as follows using VCFtools 1.1.14 (http://vcftools.sourceforge.net/index.html). To remove sites in the regions of transposable elements, a gff file (https://urgi.versailles.inra.fr/download/oak/Qrob_PM1N_refTEs.gff.gz) was obtained and transformed to a bed file.

First, we selected biallelic sites mapped to the 12 chromosomes other than the regions of transposable elements, without indels, with < 0.5 proportion of missing genotypes, > 0.05 minor allele frequency, ≥ 30 quality values. We removed genotypes with < 3 depth.

```
vcftools --vcf input.recode.vcf --chr Qrob_Chr01 --chr Qrob_Chr02 --chr
  Qrob_Chr03 --chr Qrob_Chr04 --chr Qrob_Chr05 --chr Qrob_Chr06 --chr
  Qrob_Chr07 --chr Qrob_Chr08 --chr Qrob_Chr09 --chr Qrob_Chr10 --chr
  Qrob_Chr11 --chr Qrob_Chr12 --exclude-bed Qrob_PM1N_refTEs.bed --remove-
  indels --max-missing 0.5 --maf 0.05 --min-alleles 2 --max-alleles 2 --minQ
  30 --minDP 3 --recode --recode-INFO-all --out raw
```

We confirmed the proportion of missing genotypes in each sample.

```
vcftools --vcf raw.recode.vcf --missing-indv
```

We removed samples with > 30% missing genotypes (X are this sample names) and sites with < 16 mean depth.

```
vcftools --vcf raw.recode.vcf --remove-indv X --min-meanDP 16 --recode --
  recode-INFO-all --out sel
```

Next, we filtered sites using vcffilter in vcflib (https://github.com/vcflib/vcflib) as follows.

```
vcffilter -s -f "AB > 0.25 & AB < 0.75 | AB < 0.01" sel.recode.vcf >
  fil1.recode.vcf
vcffilter -s -f "SAF / SAR > 100 & SRF / SRR >100 | SAR / SAF > 100 & SRR /
  SRF > 100" fil1.recode.vcf > fil2.recode.vcf
vcffilter -f "MQM / MQMR > 0.9 & MQM / MQMR < 1.1" fil2.recode.vcf >
  fil3.recode.vcf
vcffilter -s -f "PAIRED > 0.05 & PAIREDR > 0.05 & PAIREDR / PAIRED > 0.25 &
  PAIREDR / PAIRED < 1.75 | PAIRED < 0.05 & PAIREDR < 0.05" fil3.recode.vcf >
  fil4.recode.vcf
vcffilter -f "QUAL / DP > 0.25" fil4.recode.vcf > fil5.recode.vcf
```

We removed sites with > 100 mean depth because these sites may be multi-copy genes.

```
vcftools --vcf fil5.recode.vcf --max-meanDp 100 --recode --recode-INFO-all --
  out fil6
```

We removed genotypes with < 8 depth and selected sites with < 0.05 proportion of missing genotypes.

```
vcftools --vcf fil6.recode.vcf --minDP 8 --max-missing 0.95 --recode --recode-
  INFO-all --out fil7
```

Finally, we removed sites deviated ($P < 0.01$) from the Hardy-Weinberg equilibrium in each population.

```
vcftools --vcf fil7.recode.vcf --keep QcIgroup.txt --recode --recode-INFO-all
  --out fil7QcI
vcftools --vcf fil7QcI.recode.vcf --hwe 0.01 --recode --recode-INFO-all --out
  out1QcI
bgzip fil8QcI.recode.vcf; tabix -p vcf fil8QcI. recode.vcf.gz
```

We done these commands for each of inland *Qc*, coastal *Qc*, and coastal *Qd* populations. Then, we merged these outputs.

```
vcf-merge out1QcI.recode.vcf.gz out1QcC.recode.vcf.gz out1QdC.recode.vcf.gz >
  out1.recode.vcf
```

We removed sites with > 0.1 estimated frequency of null alleles using GBStools (https://github.com/cooketho/gbstools).

```
polymorphism_test.py -i out1.recode.vcf -o out1.recode.scored.vcf
grep -v DFreq=nan out1.recode.scored.vcf > out2.recode.scored.vcf
vcffilter -s -f "DFreq < 0.1" out2.recode.scored.vcf > out3.recode.vcf
```

To avoid including closely linked sites in the same pair-end reads, we thinned sites so that no two sites were within 1 kb from one another.

```
vcftools --vcf out3.recode.vcf --thin 1000 --recode --recode-INFO-all --out
  out4
```

We obtained genotypes at variable ddRAD sites.

```
vcf-to-tab < out4.recode.vcf > out4.recode.vcf.tab
```

Note S4. Patterson's *D* with ambiguous derived alleles

We supposed that following two cases 1 and 2 occurred equally (at 0.5 probability); case 1: non-reference alleles are derived, and case 2: reference alleles are derived. For each site *i*, we randomly selected either the case 1 or the case 2.

In the case 1, the allele in the *Q. robur* reference sequence is ancestral. Thus, q_{i4} (the frequency of non-reference allele in the outgroup population) follows a beta probability-density distribution with parameter values, 1 and 2. This distribution indicates that the probability is 0 when $q_{i4} = 1$ and that the probability increases linearly as q_{i4} decreases to 0. It is the posterior probability after the single observation under a non-informative prior. Therefore,

$q_{i4} \sim \mathrm{Beta}(1, 2)$,

$C_{\mathrm{ABBA}}[i] = (1 - q_{i1})\, q_{i2}\, q_{i3}(1 - q_{i4})$, $C_{\mathrm{BABA}}[i] = q_{i1}(1 - q_{i2})q_{i3}(1 - q_{i4})$,

where q_{i1} is the frequency of a non-reference allele at site *i* in inland *Qc* (non-introgressed population), q_{i2} is that in coastal *Qc* (putatively introgressed recipient population), q_{i3} is that in coastal *Qd* (donor population), and *n* is the number of sites in the whole genome or each chromosome.

In the case 2, the allele in the *Q. robur* reference sequence is derived. Thus, q_{i4} follows a beta distribution with parameter values 2 and 1. This distribution indicates that the probability is 0 when $q_{i4} = 0$ and that the probability increases linearly as q_{i4} increases to 1. Therefore,

$q_{i4} \sim \mathrm{Beta}(2, 1)$,

$C_{\mathrm{ABBA}}[i] = q_{i1}(1 - q_{i2})(1 - q_{i3})q_{i4}$, $C_{\mathrm{BABA}}[i] = (1 - q_{i1})q_{i2}(1 - q_{i3})q_{i4}$.

We applied a random value extracted from the beta probability-density distribution to p_{i4} and conducted 1k bootstrap sampling of sites to calculate the Patterson's *D* using the random p_{i4} value. We repeated 1k ramdom sampling of p_{i4} with the bootstrapping and calculations,

$D = \sum_{i=1}^{n}(C_{\mathrm{ABBA}}[i] - C_{\mathrm{BABA}}[i]) / \sum_{i=1}^{n}(C_{\mathrm{ABBA}}[i] + C_{\mathrm{BABA}}[i])$.

Finally, we obtained 1M bootstrap samples of D values.
